# A Prognostic Model Using Immune-Related Genes for Colorectal Cancer 

**DOI:** 10.3389/fcell.2022.813043

**Published:** 2022-02-15

**Authors:** Wei Feng, Yongxin Zhang, Wenwei Liu, Xiaofeng Wang, Tianxiang Lei, Yujie Yuan, Zehong Chen, Wu Song

**Affiliations:** ^1^ Department of Gastrointestinal Surgery, The First Affiliated Hospital, Sun Yat-sen University, Guangzhou, China; ^2^ Laboratory of General Surgery, The First Affiliated Hospital, Sun Yat-sen University, Guangzhou, China; ^3^ Digestive Diseases Center, The Seventh Affiliated Hospital of Sun Yat-sen University, Shenzhen, China; ^4^ Department of Gastrointestinal Surgery, The Third Affiliated Hospital of Sun Yat-sen University, Guangzhou, China

**Keywords:** CRC, IRGSig, LASSO analysis, PCA, GDSC, TME

## Abstract

There is evidence suggesting that immune genes play pivotal roles in the development and progression of colorectal cancer (CRC). Colorectal carcinoma patient data from The Cancer Genome Atlas (TCGA) and the Gene Expression Omnibus (GEO) were randomly classified into a training set, a test set, and an external validation set. Differentially expressed gene (DEG) analyses, univariate Cox regression, and the least absolute shrinkage and selection operator (LASSO) were used to identify survival-associated immune genes and develop a prognosis model. Receiver operating characteristic (ROC) analysis and principal component analysis (PCA) were used to evaluate the discrimination of the risk models. The model genes predicted were verified using the Human Protein Atlas (HPA) databases, colorectal cell lines, and fresh CRC and adjacent tissues. To understand the relationship between IRGs and immune invasion and the TME, we analyzed the content of immune cells and scored the TME using CIBERSORT and ESTIMATE algorithms. Finally, we predicted the potential sensitive chemotherapeutic drugs in different risk score groups by the Genomics of Drug Sensitivity in Cancer (GDSC). A total of 491 IRGs were screened, and 14 IRGs were identified to be significantly related to overall survival (OS) and applied to construct an immune-related gene (IRG) prognostic signature (IRGSig) for CRC patients. Calibration plots showed that nomograms have powerful predictive ability. PCA and ROC analysis further verified the predictive value of this fourteen-gene prognostic model in three independent databases. Furthermore, we discovered that the tumor microenvironment changed significantly during the tumor development process, from early to middle to late stage, which may be an essential factor for tumor deterioration. Finally, we selected six commonly used chemotherapeutic drugs that have the potential to be useful in the treatment of CRC. Altogether, immune genes were used to construct a prognosis model for CRC patients, and a variety of methods were used to test the accuracy of this model. In addition, we explored the immune mechanisms of CRC through immune cell infiltration and TME in CRC. Furthermore, we assessed the therapeutic sensitivity of many commonly used chemotherapeutic medicines in individuals with varying risk factors. Finally, the immune risk model and immune mechanism of CRC were thoroughly investigated in this paper.

## Introduction

Colorectal cancer (CRC) is the third most common cancer and the fourth leading cause of cancer-related deaths worldwide. In many low-income and middle-income countries, the incidence and mortality rates of CRC are rising rapidly, where the incidence rate in developed countries tends to be stable or decreasing (Arnold et al., 2017; Thanikachalam and Khan, 2019). Early diagnosis and treatment strategies are essential for improving the prognosis of patients with CRC. However, there is a lack of efficient immune genetic markers and prognosis prediction models, and most patients are detected at a late stage. As a result, it is critical to develop novel biomarkers for the noninvasive diagnosis of CRC (Deng et al., 2018; Fayazfar et al., 2019). In recent years, it has been reported that TP53, Ras mutations, and abnormal expression of TROP2, ABCG2, and HIF-1 genes play key roles in the malignant transformation of CRC. The mechanisms behind CRC development and progression are still not fully understood (Hall et al., 2018; Wei et al., 2020).

Immunotherapy has ushered in a new era of cancer therapies. Precision medicine plays a major role in the advancement of these therapies. Recently, the immune checkpoint inhibitors (ICIs), including CTLA-4 and PD-1/PD-L1 inhibitors, have become a breakthrough in cancer therapy, which has aroused great enthusiasm in tumor immunotherapy research (Llosa et al., 2015). In recent years, the U.S. Food and Drug Administration (FDA) has authorized the combination of navuzumab and pembrolizumab to treat advanced melanoma, advanced renal cell carcinoma, and MSI-H/dMMR type advanced CRC. Immunotherapy, unlike other therapies for metastatic cancers, has proved to have a long-term response in patients, enhancing its attractiveness as a powerful cancer therapy (Le et al., 2017; Ganesh et al., 2019). However, the objective response rate to ICIs is not ideal for many patients who experience drug resistance with time and who even become what is termed as “hyperprogressors” (Gao et al., 2016; Zaretsky et al., 2016; Kato et al., 2017). Therefore, the identification of novel biomarkers predicting the efficacy of immunotherapy and the use of chemotherapy-sensitive drugs for patients with different risk factors is crucial.

The study presented here emphasizes the importance of fourteen essential immune genes in developing a predictive model for CRC. This not only gives novel potential prognostic indicators and therapeutic targets, but it also enriches our understanding of the tumor immune microenvironment status, providing guidance for immunotherapy and the selection of sensitive chemotherapeutic drugs.

## Materials and Methods

### Data Collection

**FIGURE 1:**
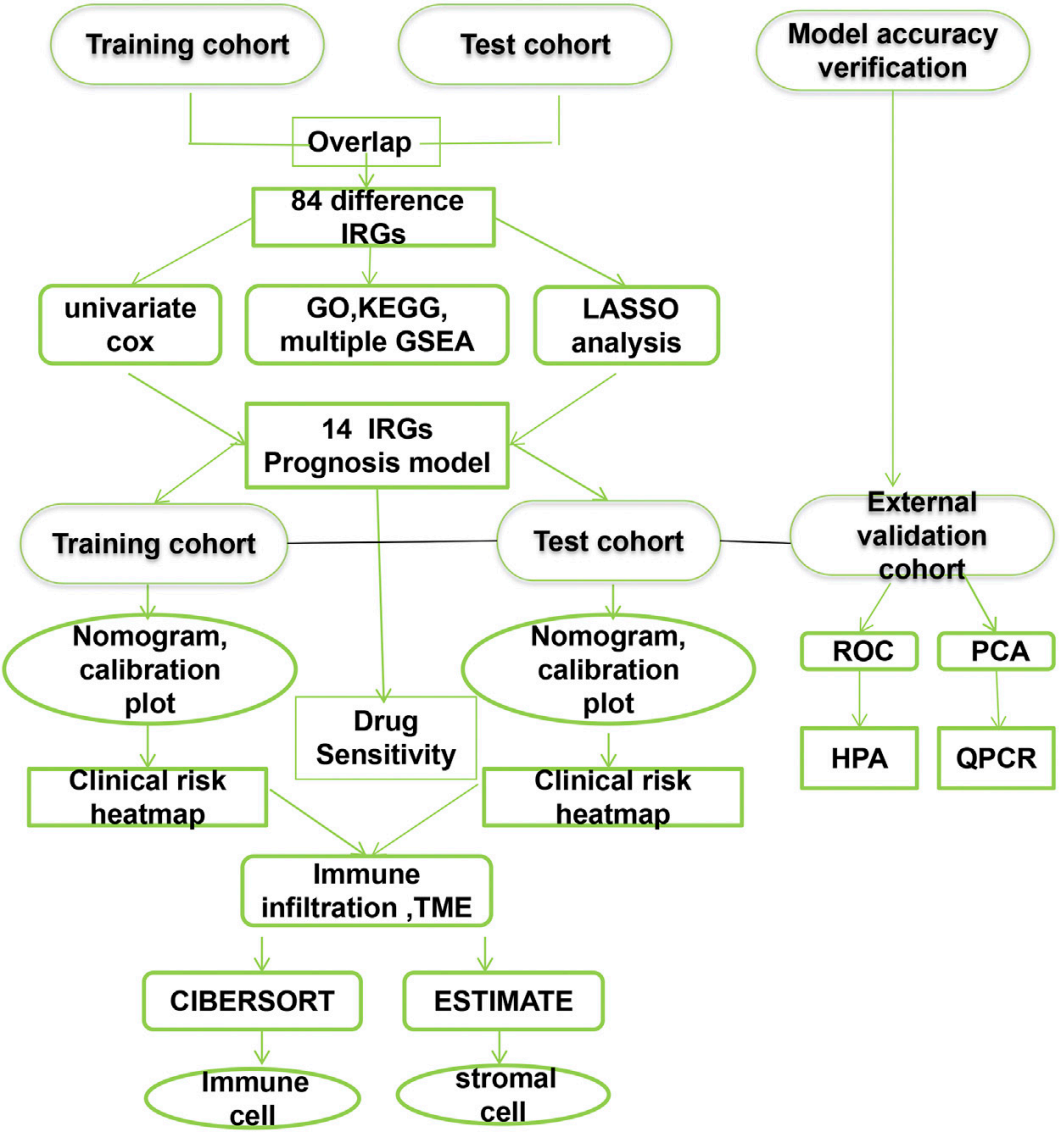
The flow chart of the analytical process.

Gene expression FPKM data for 554 CRC and 48 adjacent normal CRC tissues and clinical data for CRC patients were obtained from the TCGA database (https://portal.gdc.cancer.gov/). Finally, DEGs were identified using limma (log|fold change (FC)| > 1.0 and *p*-value < 0.05) for train analysis. Two mRNA expression datasets from GSE17538and GSE29621 (including 242 CRC samples) based on platform GPL570 were selected and acquired from the GEO database (https://www.ncbi.nlm.nih.gov/geo) as a test dataset. The GSE39582 dataset, which included a gene expression profile of 566 cases, served as an external validation dataset. Supplementary Table S1 summarized the clinicopathological characteristics of patients with colorectal cancer in the training, testing, and validation dataset. If more than one expression file had matched patients, the mean value with the highest mean value was used. Overlapping DEGs were chosen for further study. Immune-related genes (IRGs) were selected for establishing prognostic models. Patients without events and missing follow-up time were excluded to avoid bias. The entire analysis process of this research is shown in Figure 1.

### Univariate Cox Regression and LASSO Analysis in Prognostic Model Building

To identify prognostic genes, univariate Cox regression analysis of OS was performed for every single gene. IRGs of survival-related modules with *p* < 0.03 were identified as survival-related IRGs and further applied to LASSO analysis, a widely used machine learning algorithm regression for discerning prognostic risk signatures. By minimizing the *p*-value of the log-rank test, an optimal cutoff value was calculated, and the risk score for each sample in the training cohort and the test cohort was calculated. Finally, the survival differences between high- and low-risk groups in each cohort were evaluated by Kaplan–Meier curves. For clinical factors and risk scores, univariate and multivariate independent prognostic analyses were done, and a prognostic nomogram was created. The “RMS” R package was used to draw a nomogram to predict the possibility of OS, and a nomogram calibration chart was used to verify the accuracy of the ideal model. A risk plot was used to show the risk score of each sample. Independent analysis was performed on clinical and prognostic factors.

### Biological Processes and Pathways of Overlapping DEGs

The potential biological functions of overlapping DEGs were explored using Gene Ontology (GO) terms; Kyoto Encyclopedia of Genes and Genomes (KEGG) pathway enrichment analyses were performed by the cluster Profiler package. Functional categories with a *p* value < 0.05 were considered as significant pathways. GSEA was used to determine the biological pathways with rich differences between the high-risk scoring group and the low-risk scoring group. Selecting the top five pathways was for multivariate GSEA.

### Prognostic Model Evaluation

A ROC curve was constructed using the survival ROC R package to predict the prognosis of the model in train cohorts, test cohorts, and external validation cohorts. In the three cohorts, PCA was used to analyze the differences between the models in distinguishing high-risk from low-risk patients. Immunohistochemistry data from the HPA database confirmed the expression of these model genes in CRC. Moreover, the AUC values of IRGSig and three published gene prognosis models were compared, which used the same TCGA dataset.

### Tissue Samples, Cell Culture, RNA Extraction, and Quantitative Real-Time PCR

From September 2019 to December 2020, colorectal cancer tissues and paired adjacent noncancerous tissues of 62 patients were collected in the First Affiliated Hospital of Sun Yat-sen University. Our study was approved by the Ethics Committee of the First Affiliated Hospital of Sun Yat-Sen University. Before RNA extraction, the samples were stored in liquid nitrogen. Human normal colonic epithelial cells (HCoEpiC) and CRC cell lines (HCT116, DLD-1, SW480, LOVO, CaCo2, HT29) were obtained from American Type Culture Collection (ATCC, Manassas, VA, United States). Cells were cultured using DMEM (Gibco, CA, United States) containing 10% fetal bovine (Gibco, CA, United States), 100 U/ml penicillin, and 100 mg/ml streptomycin (Invitrogen, Carlsbad, CA, United States). All cell lines were cultured at 37°C with 5% CO_2_. Trizol reagent was used to extract total RNA from tissues and cell lines (Invitrogen, Carlsbad, CA). Quantitative real-time PCR (qPCR) was performed on the light cycle 480 real-time PCR platform (Merlan Roche, France) to measure gene expression. The qRT-PCR primer sequences are provided in Table 1. The relative quantification values for RNA were calculated by the 2^−ΔΔCt^ method.

**TABLE 1:** The primers of genes.

Genes	Forward primers	Reverse primers
CCL28	TGC​ACG​GAG​GTT​TCA​CAT​CAT	TTG​GCA​GCT​TGC​ACT​TTC​ATC
GNAI1	TTA​GGG​CTA​TGG​GGA​GGT​TGA	GGT​ACT​CTC​GGG​ATC​TGT​TGA​AA
STC1	AGG​TGC​AGG​AAG​AGT​GCT​ACA	GAC​GAC​CTC​AGT​GAT​GGC​TT
SPP1	CTC​CAT​TGA​CTC​GAA​CGA​CTC	CAG​GTC​TGC​GAA​ACT​TCT​TAG​AT
VIP	TGA​GGG​AGC​AAA​TGA​ACC​TGA	GCA​GAA​AGT​TGA​CCC​AAG​AGT​TT
CHP2	GTT​CTC​CGT​CTG​ATG​GTT​GGG	ACT​TGG​TGA​ACT​CCA​CGA​AGG
RETNLB	CCA​TTT​CCT​GAG​CTT​TCT​GG	AGC​ACA​TCC​AGT​GAC​AAC​CA
GAPDH	ACA​ACT​TTG​GTA​TCG​TGG​AAG​G	GCC​ATC​ACG​CCA​CAG​TTT​C

### Cibersort and Estimate Algorithm Estimations

The CIBERSORT package was applied to normalize gene expression data and infer the relative proportions of 22 infiltrating immune cell types, with 1,000 permutations and *p* < 0.05. To compare differences in immune cell subtypes in the high-risk and low-risk score groups, Wilcox test was employed, and the results were displayed using a radar map. Pearson correlation coefficients were used to calculate the associations between the 22 immune cells. The ESTIMATE package was used to investigate the relationship between the risk score and TME.

### Prediction of Potential Sensitive Chemotherapeutic Drugs

In the training set, the pRRophetic R package was used to calculate the drug sensitivity differences of colorectal cancer patients between the high-risk score group and the low-risk score group to 5-FU, acitinib, bleomycin, dabrafenib, docetaxel, and cytarabine based on GDSC (www.cancerRxgene.org). pRRophetic is a popular machine learning algorithm, which is extensively utilized in medical studies (Liu et al., 2021b; Liu et al., 2021c; Liu et al., 2022a; Liu et al., 2022b). The half maximum inhibitory concentration (IC50) was used as the comparison index.

### Statistical Analysis

Data were analyzed with R version 3.6.2. Kaplan–Meier survival analysis was managed to evaluate the relationship between IRG biomarkers and survival outcomes using the R survival package. The optimal cutoff value was used to distinguish the high- and low-risk groups. LASSO analysis was performed using the “glmnet” package. Unless otherwise noted, significance was denoted as *p* < 0.05.

## Results

### Generation of an IRG Predictive Model

**FIGURE 2:**
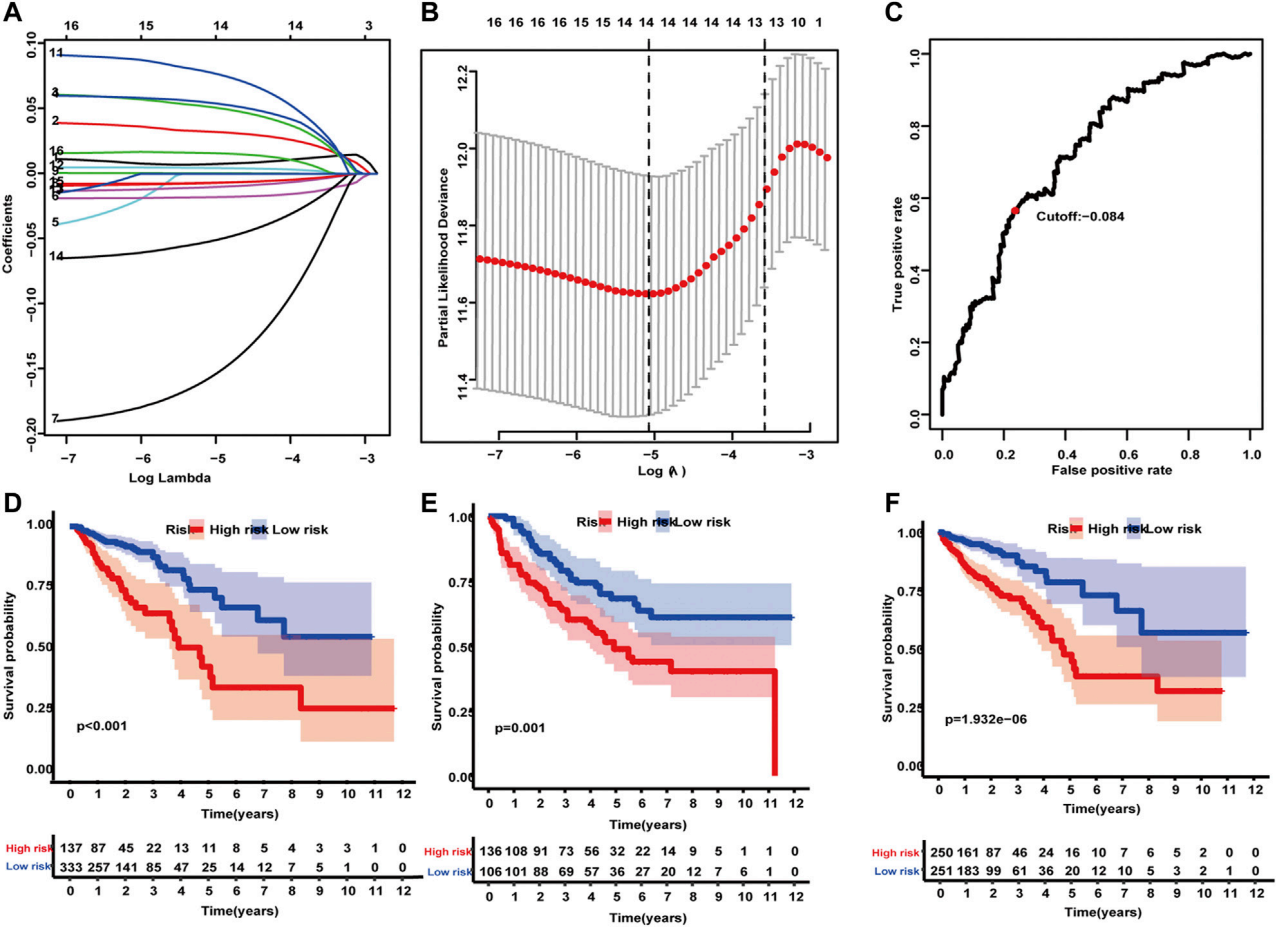
Construction and validation of an IRG predictive model. **(A)** Tenfold cross-validation for tuning parameter selection in the LASSO model using minimum criteria. **(B)** LASSO coefficient profiles of the 17 IRGs. A vertical line was drawn at the value selected using 10-fold cross-validation, where the optimal lambda resulted in 14 nonzero coefficients. **(C)** The optimal cutoff value was determined. **(D–F)** The Kaplan–Meier method applying the two-sided log-rank test was performed to estimate differences in OS between high- and low-risk groups in the training, test, and validation cohorts.

A total of 6,526 differentially expressed genes (DEGs) (4,437 upregulated and 2,089 downregulated) were identified in the TCGA dataset. In all, 493 IRGs were found to be differentially expressed in CRC vs normal tissues. Two datasets yielded 84 overlapping IRGs. IRGs expressed with a median absolute deviation (MAD) less than 0.5 were removed to reduce outliers. A heat map and volcano plot revealed that CRC samples can be distinguished from the normal samples according to differentially expressed levels of genes (Supplementary Figures.S1,2). Univariate Cox regression analysis was used to analyze the relationship between the 84 IRGs and OS. A total of 17 IRGs were significantly associated with the OS of CRC patients (*p* < 0.03), and LASSO analysis identified 14 IRGs as candidate prognostic factors (Figures 2A–C). The 14 candidate IRGs are listed in Table 2. CRC patients from the training, test, and validation cohorts were separated into low-risk and high-risk groups using an optimal cutoff value (cutoff = -0.084). The Kaplan–Meier curves showing survival differences between the two groups were plotted. Results showed that patients in the low-risk group had better OS in the training cohort (*p* < 0.001; Figure 2D **)** and the test cohort (*p* = 0.001; Figure 2E). Similar results were observed for the external validation cohort (*p* < 0.001; Figure 2F).

**TABLE 2:** Up- and downregulation of 14 differentially expressed IRGs in the model.

IRGs	Gene symbol			
Up	PROCR	STC1	INHBA	ESM1
	SPP1	CXCL3	GRP	
Down	CCL28	GNAI1	NR3C2	CCL11
	RETNLB	VIP	CHP2	

### Relationship Between Clinical and Pathological Risk Factors

**FIGURE 3:**
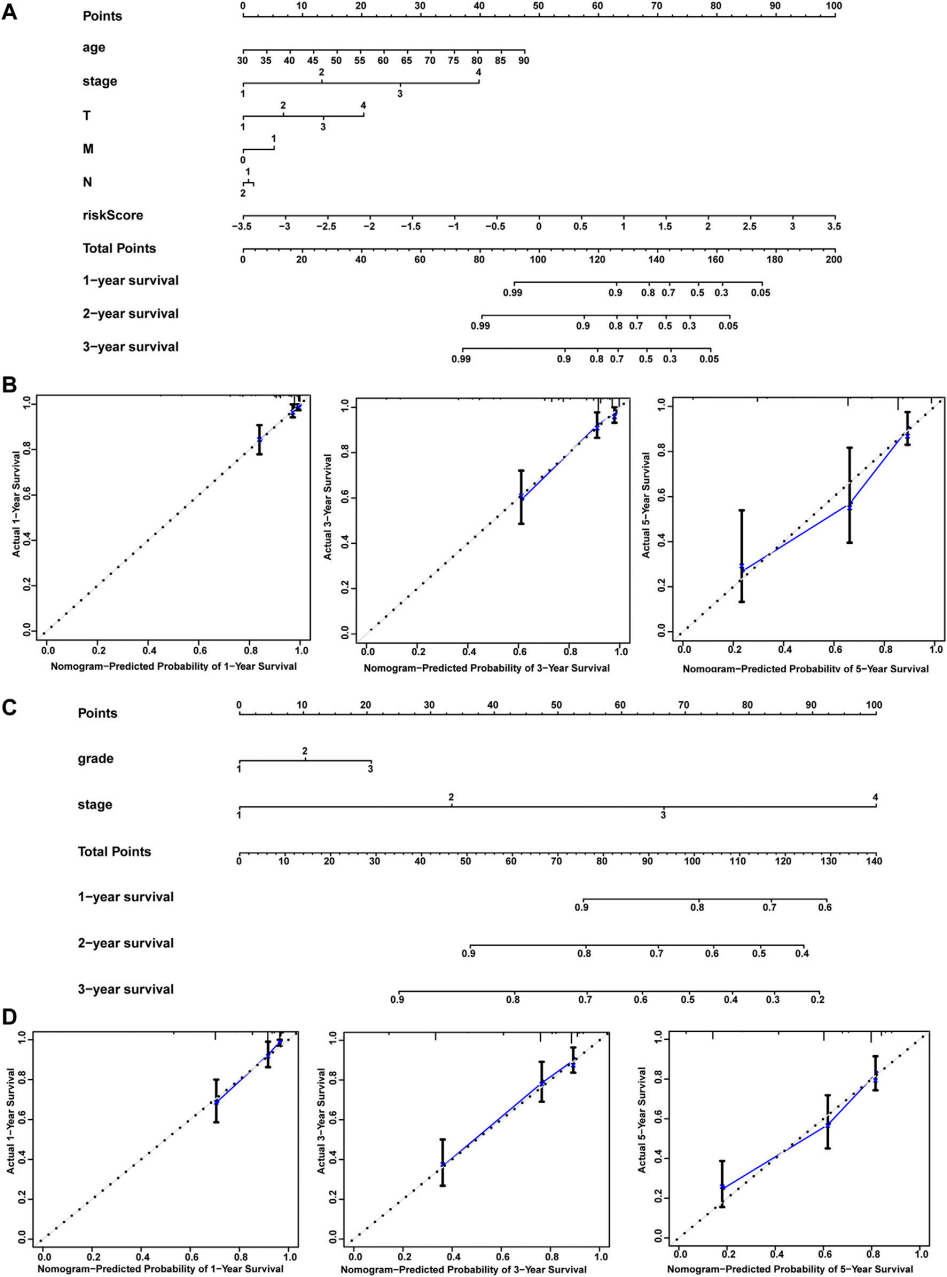
Nomograms and calibration plots. Nomogram **(A–C)** and calibration plots **(B–D)** for predicting OS at 1 year, 3 years, and 5 years for CRC patients in the training and test cohorts.

Univariate and multivariate Cox regression analyses were used to identify the prognostic association for clinical and pathological risk factors. Several clinicopathologic factors, such as stage, age, gender, and histological grade type, were incorporated into these analyses. Univariate and multivariate results exhibited in Table 3 revealed that the immune prognostic model acts as an independent factor for predicting the prognosis of CRC patients in the training and test cohorts. For the sake of applying the prognosis model, we established a nomogram for predicting CRC prognosis based on the LASSO analysis in the training and test cohorts (Figures 3A,C). The nomograms incorporated patient age, tumor stages, T, N, M, and risk score. Total points were obtained by adding the scores for each factor to predict the 1-, 3-, and 5-year survival rates for CRC patients. Calibration plots indicated that the nomograms showed accuracy compared with an ideal model both in the training and validation sets (Figures 3B,D). In summary, these models were demonstrated to be reliable tools for predicting the prognosis both in the training and test cohorts.

**TABLE 3:** Univariate and multivariate Cox regression analysis of the IRGSig and clinicopathologic factors in the training and test cohorts.

		Univariate analysis				Multivariate analysis			
	Id	Hr	HR.95L	HR.95H	Pvalue	Hr	HR.95L	HR.95H	Pvalue
Training set	age	1.038	1.013	1.063	0.003	1.055	1.028	1.083	0.000
	gender	1.084	0.660	1.780	0.750	1.019	0.614	1.692	0.941
	stage	3.079	2.273	4.169	0.000	2.461	0.968	6.257	0.059
	T	3.705	2.224	6.173	0.000	1.588	0.888	2.838	0.119
	M	6.629	4.019	10.933	0.000	1.428	0.411	4.956	0.575
	N	2.399	1.796	3.204	0.000	0.945	0.568	1.572	0.826
	riskScore	3.505	2.500	4.912	0.000	2.647	1.868	3.751	0.000
Test set	gender	1.223	0.809	1.850	0.340	0.936	0.610	1.437	0.763
	grade	1.712	1.116	2.627	0.014	1.271	0.812	1.989	0.295
	stage	2.709	2.084	3.520	0.000	3.154	2.324	4.281	0.000
	riskScore	7.695	2.416	24.511	0.001	14.981	4.432	50.641	0.000

### Tumor IRG Landscapes and Genomic Associations

**FIGURE 4:**
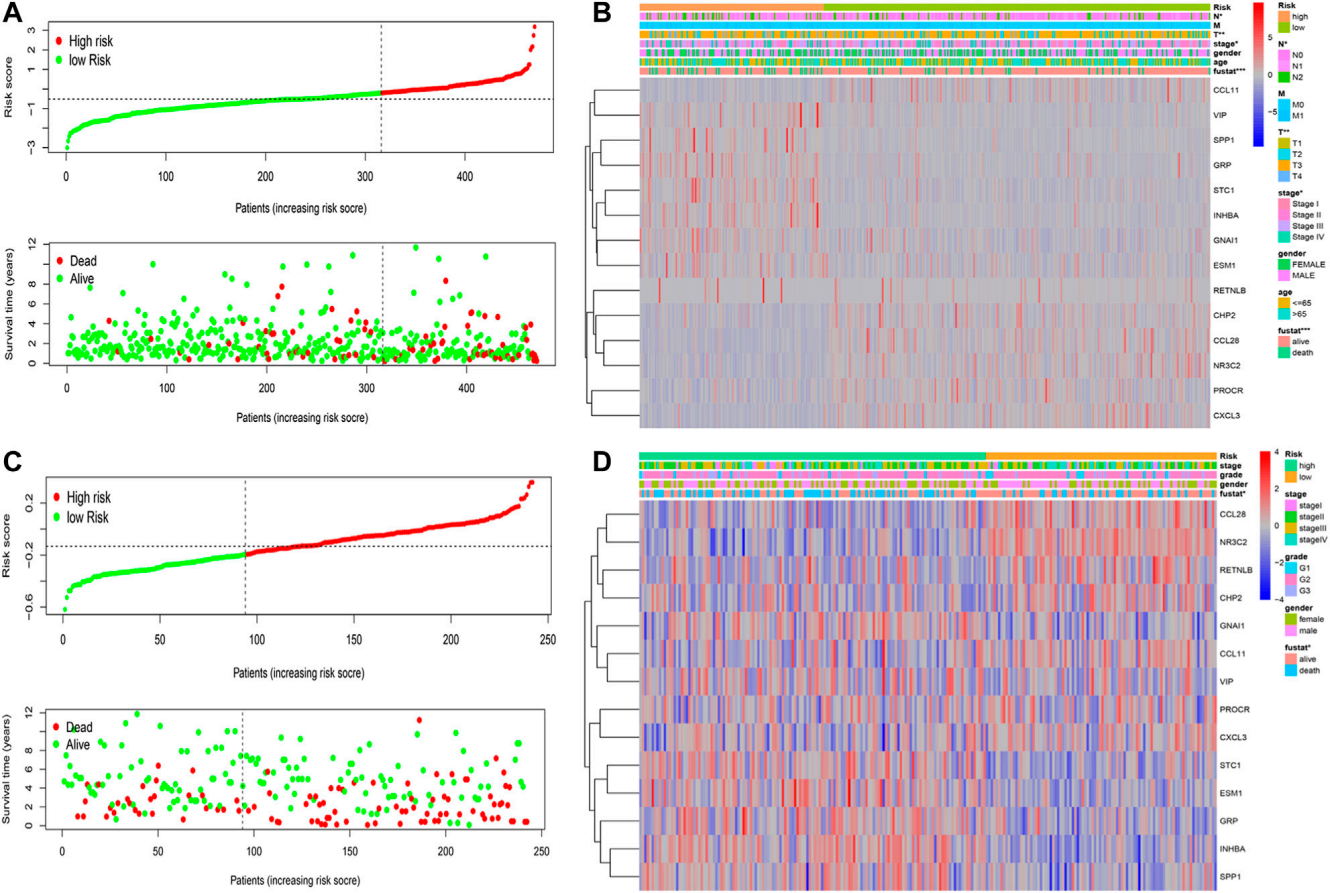
Risk IRG landscapes. **(A–C)** Risk score distribution, survival status, and duration of CRC patients in the training and test cohorts **(B–D)** Risk score analysis of the 14 IRGs prognostic model and clinical characteristics demonstrated by a heatmap for the training and test cohorts.

The risk scores of the patients in the two datasets were used to rank them. A dot plot was produced based on their survival status (Figures 4A–C), demonstrating that the low-risk group had a longer OS time. Correlations between the IRG prognostic model, clinical characteristics, risk score, as well as the expression of the 14 IRGs were evaluated. The model cohort heatmap (Figure 4B) reveals the expression of model genes in the high- and low-risk groups. Survival status, stage, T stage, and N stage were correlated with risk value (*p* < 0.05). In the test cohort heatmap (Figure 4D), the expression of model genes in the high- and low-risk groups was displayed. Fustat was related to risk value (*p* < 0.05). These data revealed significant differences in the immune landscape between low-risk and high-risk patients in the training and test cohorts.

### Functional Enrichment Analysis

**FIGURE 5:**
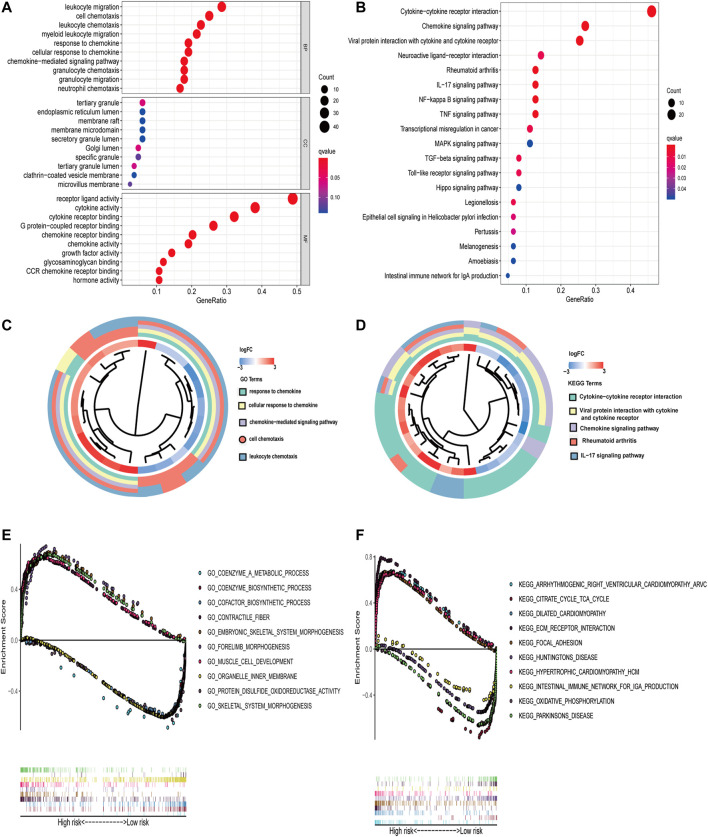
Enrichment analysis of GO and KEGG. **(A–C)** GO and **(B–D)** KEGG enrichment analysis for 84 overlapping DEGs; terms with *p* < 0.05 were believed to be enriched significantly. Several key pathways are visualized using multiple GSEA. **(E–F)** The plot of GO and KEGG enrichment analysis.

GO and KEGG analyses were performed on the 84 overlapping DEGs. The key functional categories in biological processes (BPs) found by GO analysis were leukocyte migration, cell chemotaxis, and leukocyte chemotaxis. For cellular components (CCs), the major enriched GO terms were tertiary granule and endoplasmic reticulum lumen. The most enriched molecular functions (MFs) were receptor ligand activity and cytokine activity. Among these enrichment pathways, it can be observed that the PPBP gene has the largest fold change (Figure 5A–C). According to the KEGG pathway, the overlapping DEGs were primarily engaged in Cytokinecytokine receptor interaction, Chemokine signaling pathway, and Viral protein interaction with cytokine and cytokine receptor. It can be found that the PPBP gene has the largest fold change again (Figure 5B–D). GSEA was performed to identify sets of genes representing biological processes and pathways associated with immune genes expression changes. As shown in Figures 5E,F, the top five significant GO pathways based on a risk score with FDR <0.25 included coenzyme a metabolic process, cofactor biosynthetic process, contractile fiber, coenzyme biosynthetic process, embryonic skeletal system morphogene sulfide oxidoreductase activity, and skeletal system morphogenesis. Significant Kyoto Encyclopedia of Geis, forelimb morphogenesis, muscle cell development, organelle inner membrane, protein dishes and Genomes (KEGG) pathways consisted of arrhythmogenic right ventricular cardiomyopathy arvc, citrate cycle the TCA cycle, the intestinal immune network for IGA production, dilated cardiomyopathy, ECM receptor interaction, focal adhesion, hypertrophic cardiomyopathy HCM, Huntington’s disease, oxidative phosphorylation, and Parkinson’s disease.

### Verification the IRG Prognosis Model Based on the ROC, PCA, and HPA

**FIGURE 6:**
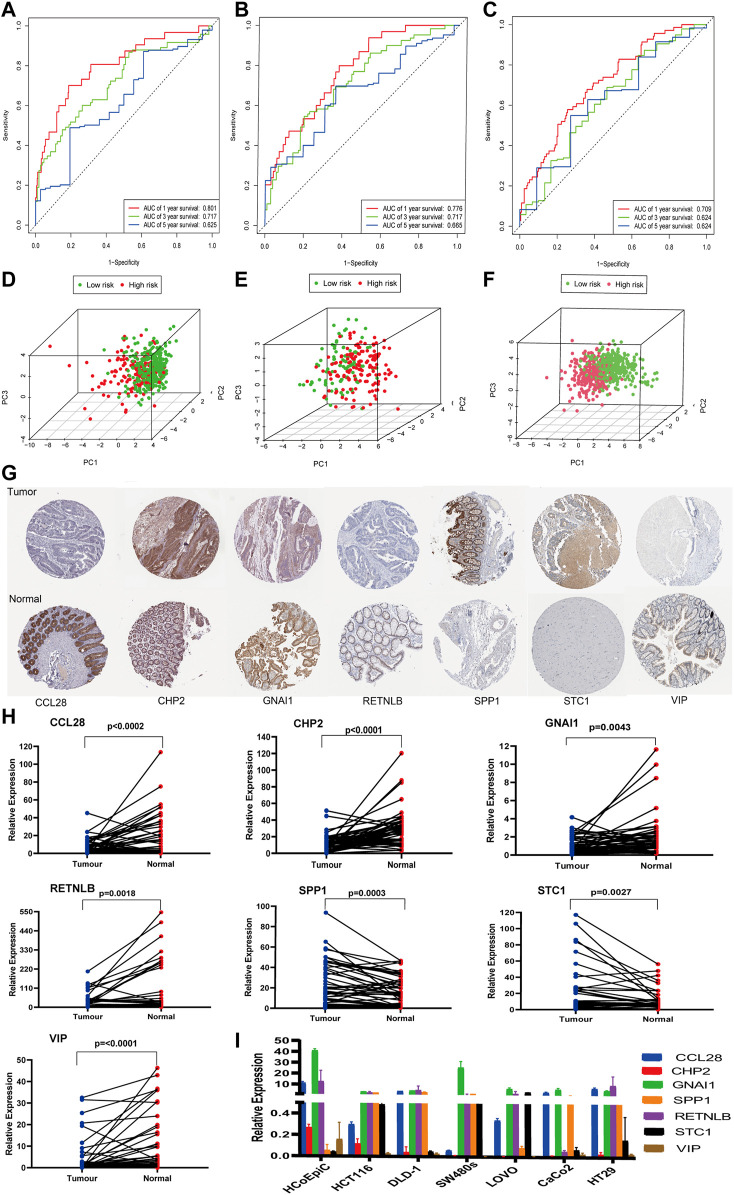
ROC analysis, PCA, HPA analysis, and qPCR. **(A–C)** ROC analysis using the risk score to predict the OS of the training, test, and validation cohorts. **(D–F)** PCA of three cohort immune-share genes is shown. **(G)** Verifying differential expression of IRGs in normal colorectal tissue and CRC using the HPA database. **(H–I)** Verification of the mRNA expression level of IRGs in fresh tissues and cell lines.

A time-dependent ROC analysis was performed to evaluate the prognostic performance of the 14 IRGs. The AUC of the training, test, and validation cohorts was 0.801, 0.776, and 0.709 after 1 year; 0.717, 0.717, and 0.624 after 2 years; and 0.625, 0.665, and 0.624 after 3 years, respectively (Figure 6A–C). Furthermore, we generated PCA graphs for training set risk genes, test set risk genes, and validation cohort test risk genes (Figures 6D–F). Green dots indicated low risk, whereas red ones indicated high risk. These genes differed in the expression between low- and high-risk patients, with the model genes having the best discrimination ability. In conclusion, PCA and ROC analysis revealed that the IRG model was effective and accurate in predicting the OS of CRC patients. Immunohistochemical results from the HPA database were utilized to further assess the expression of these model genes in CRC. All of the patient samples in this database came from the TCGA. Compared with adjacent tissues, the protein expression levels of five genes (CCL28, CHP2, GNAI1, RETNLB, and VIP) in CRC tissues decreased significantly, while the protein expression levels of SPP1 and STC1 in CRC tissues increased. In addition, these data showed significant differences in CCL28, CHP2, GNAI1, RETNLB, SPP1, STC1, and VIP between CRC and normal CRC tissues, and this trend is the same as in the model (Figure 6G).

### Verification of the mRNA Expression Level of IRGs in Clinical Tissues and Cell Lines

RNA was extracted from 62 pairs of colorectal cancer tissues and adjacent noncancer tissues, and the differential mRNA expression levels of the above seven genes were analyzed by qPCR. The mRNA levels of five genes (CCL28, CHP2, GNAI1, RETNLB, and VIP) were significantly downregulated in the CRC tissues, whereas SPP1and STC1 were elevated in the CRC tissues when compared with adjacent tissues (Figure 6H). This trend was consistent with the trend of the HPA database. In addition, the mRNA expression of the seven genes in HCoEpiC and CRC cell lines was detected by qPCR. As shown in Figure 6I, compared with HCoEpiC, the mRNA levels of five genes (CCL28, CHP2, GNAI1, RETNLB, and VIP) decreased significantly, while the expression of SPP1 and STC1 increased in CRC cell lines. The results are consistent with the results of fresh tissue validation.

### Comparison Between IRGs Signatures and Published IRGs Signatures Predicts Accuracy

**FIGURE 7:**
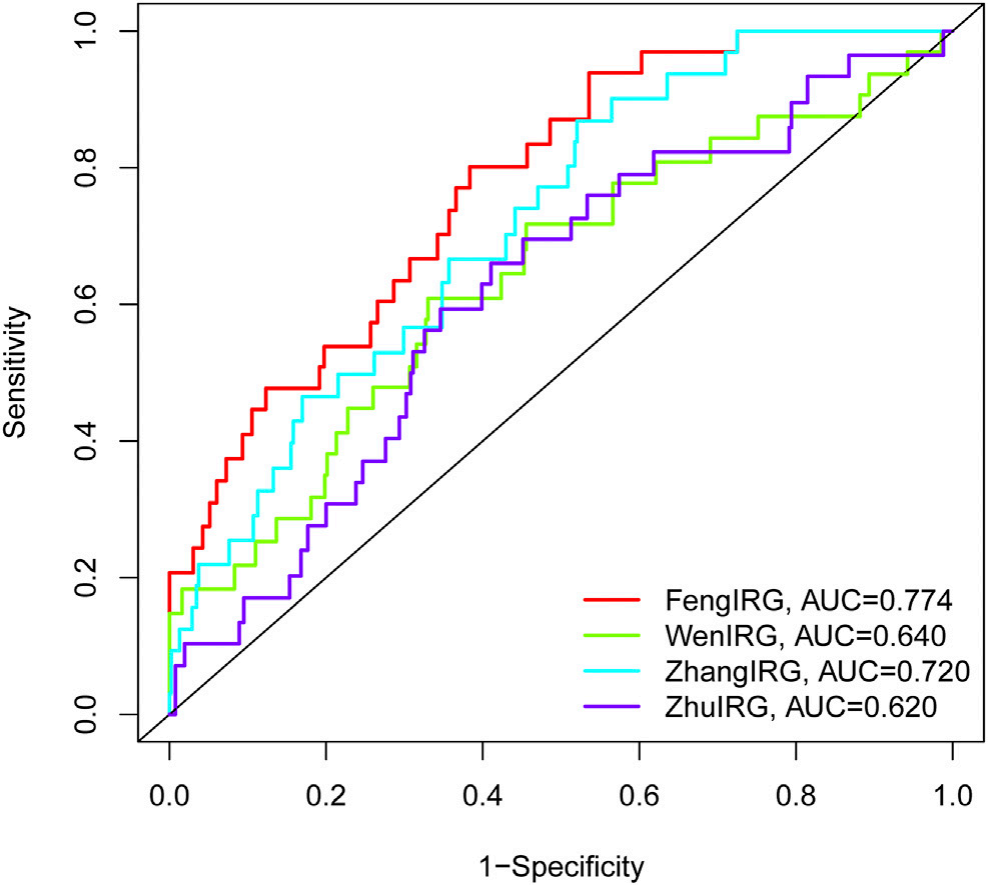
The AUC of OS for the IRGSig and three previously reported IRGs signatures at 1 year.

The AUC at 1 year OS was compared between the IRGs signature and three published IRGs signatures, which included an 8-gene signature (Wen IRGSig), a 6-gene signature (ZhangIRGSig), and a 5-gene signature (Zhu IRGSig) in the same TCGA cohort (Zhang et al., 2020b; Wen et al., 2020; Zhu et al., 2020). The AUC of the IRGs signature at 1-year OS was 0.774, which was the highest of that of Wen IRGSig (0.640), ZhangIRGSig (0.720), and Zhu IRGSig (0.620) (Figure 7). Therefore, our signature prediction accuracy was higher than the above three published IRGSig.

### Immune Cell Composition in CRC

**FIGURE 8:**
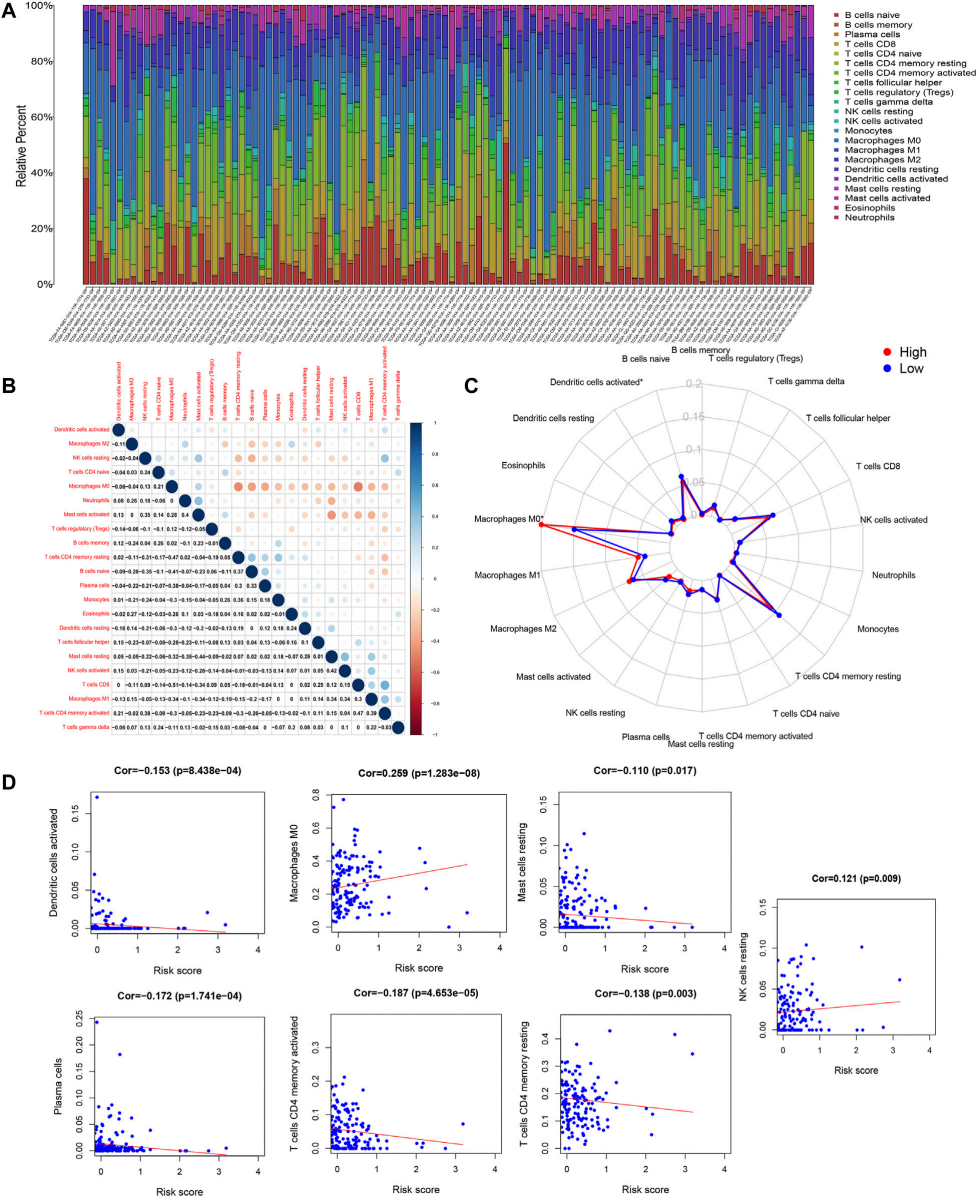
CIBERSORT results. **(A)** Estimated scores of 22 immune cell subtypes. Each bar chart presented cell proportions of each patient, and different colors represented 22 types of immune cells. **(B)** Co-expression patterns of immune cell components. **(C)** The immune cell landscape between low- and high-risk groups is shown by a radar plot. Red and blue represent high RS groups and low RS groups, respectively. The expression of immune cell types between the two groups differed. **(D)** The relationship between the risk model score and the content of seven immune cell types.

The immune cell composition was computed using CIBERSORT, a deconvolution algorithm based on normalized gene expression profiles. Figure 8A shows the proportion of 22 immune cells obtained through the CIBERSORT algorithm with *p* < 0.05. Pearson correlation analysis was used to examine significant co-expression patterns between subsets of immune cells (Figure 8B). The change of the proportion of tumor-infiltrating immune cells may be an intrinsic feature that can characterize individual differences. In addition, the proportion of tumor-infiltrating immune cells in different subsets was weakly to moderately correlated. Immune cell subtypes between high and low RS groups showed differential immune cell expression (Figure 8C). The radar plot showed expression levels of macrophages M0 and dendritic cells were significantly different between the high and low RS groups. We explored the association between the risk score model and immune cell infiltration based on the tight relationship between immunological features and CRC prognosis. Macrophages M0 and NK cells were positively associated with a risk score, as shown in Figure 8D, while dendritic cells activated, Mast cells resting, plasma cells, memory activated CD4 T cells, and CD4 memory resting T cells were negatively associated with a risk score.

### Degree of Stromal Cell Infiltration in High and Low RS Groups

**FIGURE 9:**
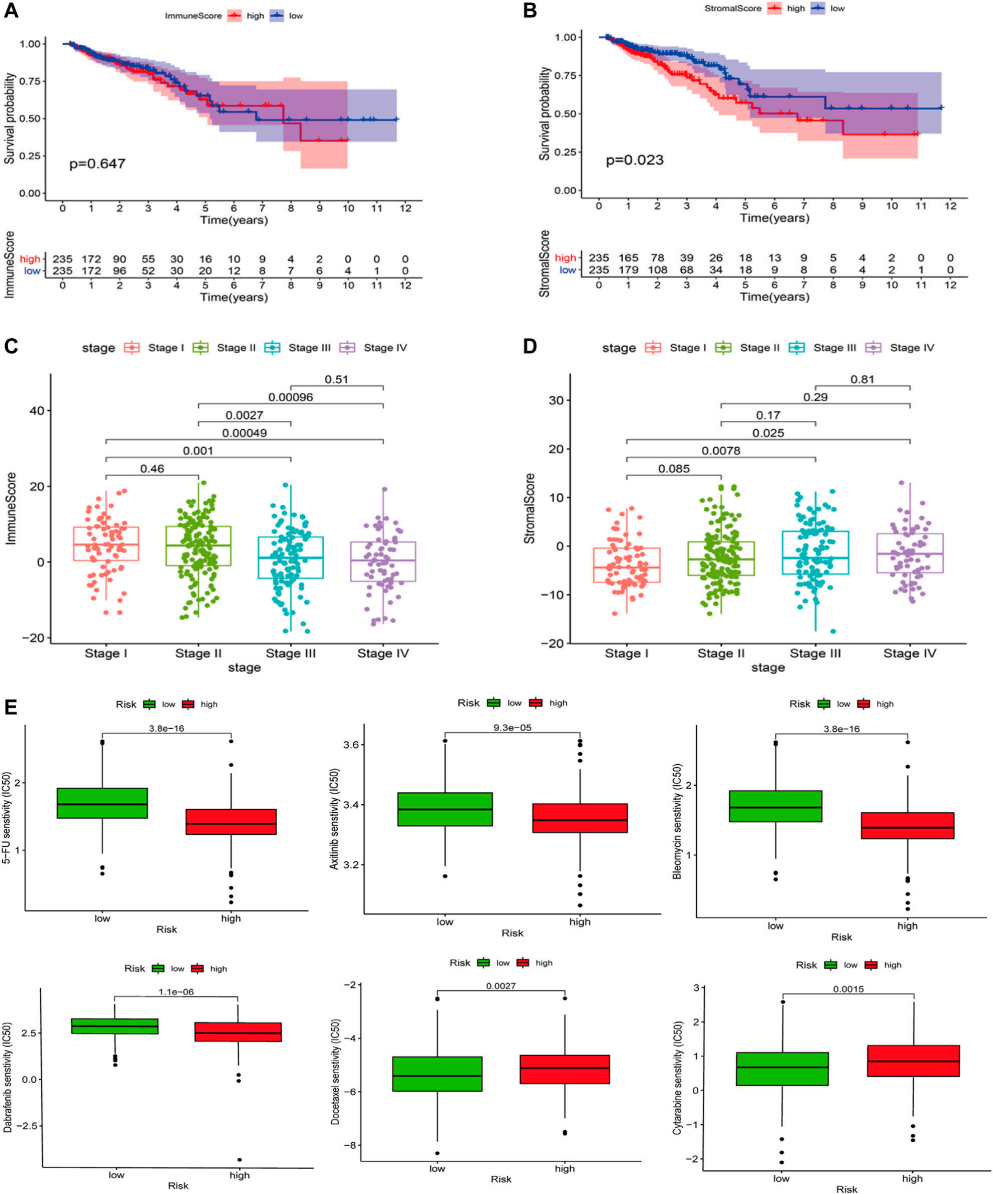
ESTIMATE results. **(A)** Association with the stromal score and the risk score. The high RS group showed a greater stromal score compared with the low RS group. **(B)** Impact of the stromal score on OS in CRC cases based on KM analysis. **(C,D)** Correlation of the stromal score and the immune score with clinicopathological stage characteristics. **(E)** The IC50 value of 5-FU, axitinib, Bleomycin, Dabrafenib, Docetaxel, and Cytarabinein in different risk score groups.

The ESTIMATE algorithm was used to calculate the stromal scores for each sample, and the Wilcoxon test was used to assess the difference in stromal cell infiltration between the low- and high-risk groups. The high RS group showed a larger stromal score compared with the low RS group. A higher stromal score group showed a shorter survival time (Figures 9A,B). There were significant differences in stromal cell scores between stage I and stage III, stage IV. Moreover, the trend was similar in immune scores (Figure 9C,D).

### Drug Sensitivity Analysis

The IC50 values of 5-FU, axitinib, Bleomycin, and Dabrafenib in the high-risk group were significantly higher than those in the low-risk group, indicating that these four chemotherapeutic drugs were more effective in a high-risk group (*p* < 0.0001, *p* < 0.0001, *p* < 0.0001). However, the IC50 values of Docetaxel and Cytarabinein were lower than those in the high-risk group, indicating that these two chemotherapeutic drugs were more effective for patients in the low-risk group (*p* < 0.0027, *p* < 0.0015) (Figure 9E).

## Discussion

CRC is considered the third most common malignant tumor and the fourth leading cause of cancer deaths worldwide (Siegel et al., 2017). Because of the important role of the immune environment in tumor progression, it is necessary to seek immune biomarkers that predict the prognosis of CRC patients, which may play a vital part in immunotherapy (Chen and Mellman, 2017). In this study, we established the survival prediction model of 14 IRGs using the CRC patient datasets in TCGA and GEO. Moreover, we combined the IRGSig score and clinicopathological features to construct a nomogram to predict the OS of CRC. The calibration curve shows that the nomograms based on IRGSig perform well in OS prediction. In addition, GO and KEGG functional analysis of DEGs and multiple GSEA indicated that some of these biological processes and signaling pathways were involved in immune and metabolic processes.

The HPA database revealed the protein expression of these model genes (CCL28, GNAI1, STC1, SPP1, RETNLB, VIP, and CHP2) in tumors and normal tissues as consistent with our analysis. The mRNA expression levels of seven IRGs in IRGSig were validated in our laboratory using freshly paired CRC and adjacent tissues from our hospital, CRC cell lines, and normal CRC epithelial cell line. In conclusion, the mRNA expression trends of TCGA, GEO, HPA, our clinical specimens, and cell lines are consistent in CRC and adjacent tissues.

Furthermore, when compared to the three previously reported signatures, our signature had the best accuracy in predicting survival. Other models that have been published have either no internal testing or no external validation. Our data were divided into a training group, a test group, and an external verification group. Each group analyzed its AUC value and PCA, which fully verified the accuracy and effectiveness of the model. At the same time, we analyzed the results of the HPA database and carried out experimental verification with fresh tissue samples and cell lines. This finding indicates that the genetic features we created were more reliable and accurate than those of previous publications. Although database analysis and *in vitro* experiments confirmed the differential mRNA and protein expression levels of these IRGs in CRC and adjacent tissues, there were no expression data in the HPA database for the other seven genes in the model, so we did not conduct *in vitro* experimental verification, which is the study’s restriction. To explore the molecular mechanism of these IRGs in CRC, further experiments *in vivo* and *in vitro* are needed.

Most IRGs were related to the occurrence and development of CRC. CCL28 in straight epithelial cells, as a chemokine, can recruit Treg lymphocytes and plays a key role in inflammation-induced tumor progression (Elemam et al., 2019). CRC cell line HCT-116 can secrete CCL28 after drug stimulation *in vitro*. The cultured supernatant can induce NK92 to dissolve tumor cells (Nguyen et al., 2013). Many CRC models have reported that CHP2, GNAI1, and RETNLB are important to core pathway genes in the progression of CRC. Several genes were identified as biomarkers affecting CRC metastasis, including ChP2, and their low expression in CRC cell lines was confirmed, compared with normal colorectal epithelial cell lines (Liang et al., 2016; Lian et al., 2020; Ma et al., 2020). SPP1 levels were found to be increased in the blood of CRC patients, and their expression was related to a poor prognosis. One of the mechanisms of SPP1 in tumor promotion was to inhibit T cell activation, which can be used as an immune checkpoint and may compensate for the role of PD-L1 in inhibiting the function of cytotoxic T lymphocytes in CRC. Using its mechanism, two monoclonal antibodies (100d3 and 103d6) were developed, which can effectively enhance the cleavage activity of tumor-specific cytotoxic T lymphocytes, kill colon tumor cells, and inhibit the growth of colon tumors *in vivo* (Klement et al., 2021). Platelet-derived growth factor (PDGF) receptor signaling is the main functional determinant of tumor-associated fibroblasts. Fibroblasts stimulated by it increase the migration and invasion of co-cultured CRC cells in an STC1-dependent manner (Pena et al., 2013). Some studies had shown that the relative expression level of the STC1 gene in cancer tissues was higher than that in adjacent normal mucosa; the high expression of STC1 was associated with a low postoperative survival rate. STC1 and other genes constitute a molecular network, which endows CRC with chemoresistance (Tamura et al., 2011; Jary et al., 2020).VIP can regulate the growth and function of various immune cells and tumor cells. Compared with normal blood cells and other mesenchymal cells, various tumor cells, including colonic adenocarcinoma, pancreatic cancer, and carcinoid, express a large number of VIP receptors (Virgolini et al., 1994).

With the emergence of cancer immunotherapy, the primary goal of treatment has shifted from cancer cells to the entire cancer microenvironment, which includes immune cells and matrix components. Addressing specific tumor features, modifying cancer’s immune setpoint, and regulating the subtle interactions between tumor and immune cells are all important strategies for enhancing patient quality of life (Reck et al., 2016; Hodi et al., 2018; Dobosz et al., 2020). Recently, many studies have shown that the TME is involved in tumor proliferation, metastasis, and drug resistance. In the TME, immune cells and stromal cells, both of which are two major nontumor components, are considered of great value in the diagnosis and prognosis of tumors (Huang et al., 2019; Tuccitto et al., 2019; Huang et al., 2020; Luo et al., 2020; Zhang et al., 2020).

Immune infiltrating cells in CRC investigated in this research included naïve B cells, CD8 T cells, M0 macrophages, M1 macrophages, and M2 macrophages. The radar map demonstrated that the high-risk group had a high density of M0 macrophages, but activated dendritic cells were lower and statistically significant. This suggests that the imbalance in M0 macrophages and dendritic cells in the high-risk group may reduce the survival of patients. Previous articles have shown that cancer cells can cooperate with macrophages to promote proliferation, migration, invasion, angiogenesis, and metastasis, which are all consistent with our findings (Huang et al., 2018; Wei et al., 2021; Xiao et al., 2021; Zhang et al., 2021).

During carcinogenesis, cell composition in the stroma changes from a subtype that protects the immune mechanisms of the body to an “auxiliary tumor cell” subtype that supports tumor cell growth, proliferation, immersion, and metastasis (40). Patients with a high stromal score had a poor prognosis in our study, and the high-risk group in the model also had a high stromal score. The scores of stromal cell and immune cell between stage I and stage III, stage IV were remarkably different, suggesting that the change of tumor microenvironment promoted the progression of tumor from early onset to middle and late stage. Immune infiltration and stromal cell analyses showed that the prognostic value of the risk model was related to the CRC immune system (Liu et al., 2021; Wang et al., 2021).

GDSC is the largest public resource for cancer cell drug sensitivity information and drug response molecular markers. At present, it contains nearly 75,000 experimental drug sensitivity data, describing the response of nearly 700 cancer cell lines to 138 anticancer drugs (Yang et al., 2013). To better guide the treatment of CRC, drug sensitivity analysis was carried out on high- and low-risk groups, and six possible drugs were identified through GDSC calculation, including 5-FU, axitinib, Bleomycin, Dabrafenib, Docetaxel, and Cytarabinein. Through our model, we can identify high- and low-risk patients from the CRC population, adopt chemotherapeutic drugs sensitive to them, and combine several sensitive drugs, which can improve the chemotherapeutic effect as well as reduce drug resistance. Therefore, these results may have more clinical guiding significance than the published literature.

In conclusion, we developed and verified an IRGSig that can predict exactly the OS and sensitivity to common chemotherapeutic drugs in patients with CRC, as well as analyze the characteristics of immune cell infiltration and tumor microenvironment, which may provide understanding into the immune mechanism and aid in the discovery of sensitive CRC therapeutic drugs.

## Data Availability

The datasets presented in this study can be found in online repositories. The names of the repository/repositories and accession number(s) can be found in the article/Supplementary Material.
