# Supplementary material for: A Prognostic Model Using Immune-Related Genes for Colorectal Cancer
Source: Front Cell Dev Biol. 2022 Feb 15;10:813043. doi: 10.3389/fcell.2022.813043 (PMC8893267; doi:10.3389/fcell.2022.813043)
Supplement: Supplementary file 1 [file DataSheet1.docx]

**Figure S1 Heatmap of significant DEGs in CRC. Blue to red represents low to high expression respectively.**


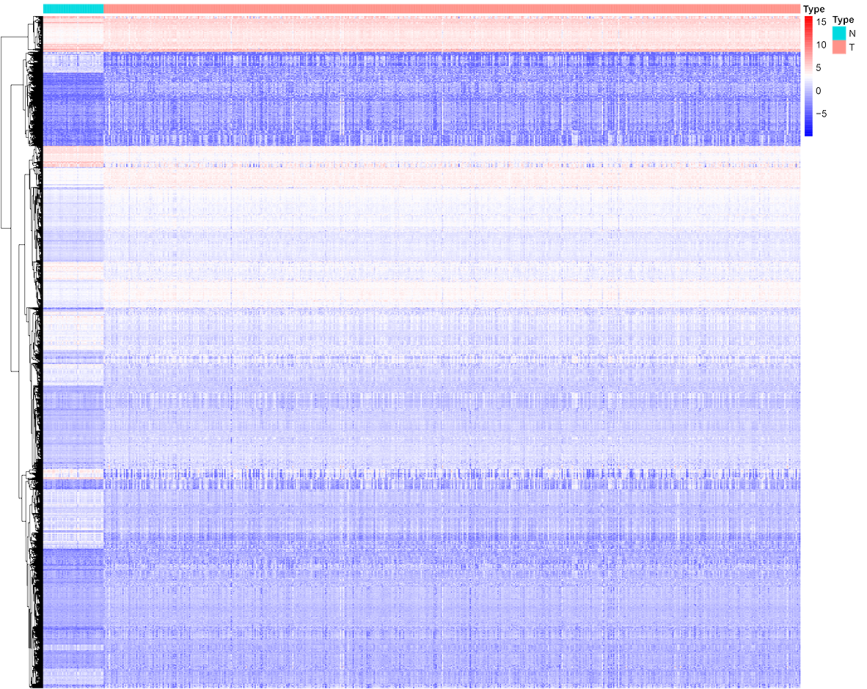


**Figure S2 A Volcano plot of DEGs. The red dots in the plot represent upregulated genes and green dots represent downregulated genes showing statistical significance.**


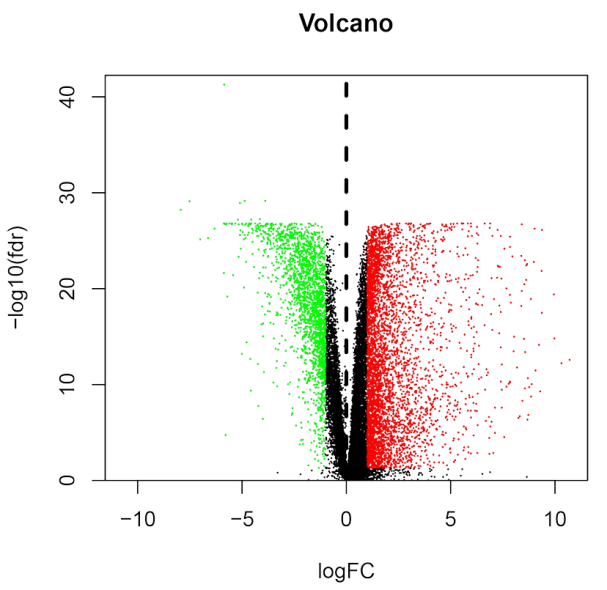


**Table S1. The clinical characteristics of CRC patients in the training, test and validation data set[n (%)].**

| **Variables** | **Training cohort (N=543)** | **test cohort (N=242)** | **Validation cohort (N=566)** |
| --- | --- | --- | --- |
| **Age** |  |  |  |
| <65 | 221（40.7） | 78(32.2) | 211（37.3） |
| ≥65 | 322（59.3） | 99(40.9) | 355（62.7） |
| Unknown |  | 65(26.9) |  |
| **Gender** |  |  |  |
| male | 291（53.6） | 135（56.2） | 310（54.8） |
| female | 252（46.4） | 106（43.8） | 256（45.2） |
| **grade** |  |  |  |
| well diff |  | 20（8.3） |  |
| mod diff |  | 185（76.4） |  |
| poorly diff |  | 37（15.3） |  |
| **Tumor invasion** |  |  |  |
| T1 | 19（3.5） |  | 15（2.7） |
| T2 | 98（18） | 8(3.3) | 45（7.9） |
| T3 | 369（68） | 52(21.5) | 367（64.8） |
| T4 | 56（10.3） | 5(2.1) | 119（21.1） |
| Unknown | 1（0.2） | 177（73.1） | 20（3.5） |
| **Metastasis** |  |  |  |
| M0 | 401（73.8） | 46（19） | 482（85.2） |
| M1 | 81（15） | 18（7.4） | 61（10.8） |
| Unknown | 61（11.2） | 176（73.6） | 23（0.4） |
| **Lymph node** |  |  |  |
| N0 | 308（56.7） | 32（13.2） | 308（54.5） |
| N1 | 132（24.3） | 25（10.3） | 134（23.6） |
| N2 | 100（18.4） | 7（2.9） | 104（18.4） |
| Unknown | 3（0.6） | 178（73.6） | 20（3.5） |
| **Pathological stage** |  |  |  |
| Stage I | 97（17.9） | 31(12.8) | 37（6.5） |
| Stage II | 193（35.5） | 79(32.6) | 264（46.7） |
| Stage III | 152（28） | 75(31) | 205（36.2） |
| Stage IV | 82（15.1） | 57(23.6) | 60（10.6） |
| Unknown | 19（3.5） |  |  |
